# Geospatial Correlation Analysis between Air Pollution Indicators and Estimated Speed of COVID-19 Diffusion in the Lombardy Region (Italy)

**DOI:** 10.3390/ijerph182212154

**Published:** 2021-11-19

**Authors:** Lorenzo Gianquintieri, Maria Antonia Brovelli, Andrea Pagliosa, Rodolfo Bonora, Giuseppe Maria Sechi, Enrico Gianluca Caiani

**Affiliations:** 1 Electronics, Information and Biomedical Engineering Department, Politecnico di Milano, 20133 Milano, Italy; lorenzo.gianquintieri@polimi.it; 2 Civil and Environmental Engineering Department, Politecnico di Milano, 20133 Milano, Italy; maria.brovelli@polimi.it; 3 Istituto per il Rilevamento Elettromagnetico dell’Ambiente, Consiglio Nazionale delle Ricerche, 20133 Milano, Italy; 4 Agenzia Regionale Emergenza Urgenza (AREU), 20124 Milano, Italy; a.pagliosa@areu.lombardia.it (A.P.); r.bonora@areu.lombardia.it (R.B.); g.sechi@areu.lombardia.it (G.M.S.); 5 Istituto di Elettronica e di Ingegneria dell’Informazione e delle Telecomunicazioni, Consiglio Nazionale delle Ricerche, 20133 Milano, Italy

**Keywords:** COVID-19, SARS-CoV-2, pollution, health geomatics, emergency medical services, correlation analysis

## Abstract

Background: the Lombardy region in Italy was the first area in Europe to record an outbreak of COVID-19 and one of the most affected worldwide. As this territory is strongly polluted, it was hypothesized that pollution had a role in facilitating the diffusion of the epidemic, but results are uncertain. Aim: the paper explores the effect of air pollutants in the first spread of COVID-19 in Lombardy, with a novel geomatics approach addressing the possible confounding factors, the reliability of data, the measurement of diffusion speed, and the biasing effect of the lockdown measures. Methods and results: all municipalities were assigned to one of five possible territorial classes (TC) according to land-use and socio-economic status, and they were grouped into districts of 100,000 residents. For each district, the speed of COVID-19 diffusion was estimated from the ambulance dispatches and related to indicators of mean concentration of air pollutants over 1, 6, and 12 months, grouping districts in the same TC. Significant exponential correlations were found for ammonia (NH_3_) in both prevalently agricultural (R^2^ = 0.565) and mildly urbanized (R^2^ = 0.688) areas. Conclusions: this is the first study relating COVID-19 estimated speed of diffusion with indicators of exposure to NH_3_. As NH_3_ could induce oxidative stress, its role in creating a pre-existing fragility that could have facilitated SARS-CoV-2 replication and worsening of patient conditions could be speculated.

## 1. Introduction

The epidemic of coronavirus disease 2019 (COVID-19), generated by the diffusion of the severe acute respiratory syndrome coronavirus 2 (SARS-CoV-2), officially started in Italy on 21 February 2020, when the first case was diagnosed in the hospital of Codogno (Lodi province), which is a small city in the south of Lombardy region, the most populated area of Italy with 10.06 million resident people (16.67% of the total 60.36 million people living in Italy). The Lombardy region was the first area in all of Europe to record an endemic outbreak of COVID-19 and became one of the most affected all over the world during the first peak, with 78,105 confirmed cases (0.78% on the total population) reached at 4 May 2020 (first lift of lockdown measure), although it is widely recognized that the actual cases might have been even ten times higher, as inferred by the recorded cases fatality rate (CFR, number of casualties/total number of diagnosed patients) set at 13.72% at 4 May 2020 compared to the estimated infection fatality rate (IFR, number of casualties/total number of infected patients) considered to be 0.5–1% [1].

The reasons for such a powerful spread of the pandemic still need to be assessed and are certainly manifold, including demographic and environmental factors. As the territory of the Lombardy region is one of the most polluted areas in Europe (https://earthobservatory.nasa.gov/images/15900/smog-in-northern-italy, accessed on 3 November 2021), it was straightforward to hypothesize that air pollution could have played a role in facilitating the diffusion of the epidemic. As a matter of fact, it is well known that air pollution is a significant cause of respiratory diseases [2,3,4,5,6,7] and has a negative impact on overall health conditions [8,9].

The hypothesis of a correlation between air pollutant levels and COVID-19 diffusion has been widely addressed by recent scientific literature. Twenty different publications on this topic were identified [8,10,11,12,13,14,15,16,17,18,19,20,21,22,23,24,25,26,27,28]: most of them (50%) were focused on Italy [10,11,12,13,14,15,16,17,18,19], whereas three were focused on the USA [8,20,21], three on China [22,23,24], one on France [25], one on England [26], and two were cross-country (one on Europe [27] and one worldwide [28]). Six of these studies considered the effects of pollution on COVID-19 casualties [12,20,21,23,25,27], seven dealt with the number of confirmed infections [10,11,13,16,17,22,24], while the remaining seven took into consideration both infections and casualties [8,14,15,18,19,26,28]. The air pollutants considered in the analysis were particulate matter (PM_2.5_ [8,12,13,14,15,16,18,20,21,22,23,24,25,26,28], and PM_10_ [8,10,11,12,15,16,17,18,22,23,24,25,26]), nitrogen dioxide (NO_2_ [8,12,13,14,16,19,20,22,24,26,27,28]), sulfur dioxide (SO_2_ [8,24]), ozone (O_3_ [11,12,19,20,24,26]), carbon oxide (CO [8,22,24]), and nitrogen oxide (NO [16,26]). The level of each pollutant was usually computed through average concentrations [8,10,12,13,14,16,18,19,20,21,22,23,24,25,26,27,28], whereas some studies (all relevant to Italy) applied, as an indirect measure of pollution level, the number of days in which the European threshold for daily average concentration was exceeded [11,15,17]; finally, other studies considered both approaches [10,12,26]. The most applied analysis method was represented by studying the correlation between air pollutant measurements and COVID-19 data, which was represented by the number of positive cases or/and casualties, relevant to different regions, considering cumulated data computed on a given time window [11,12,13,14,15,17,20,21,23,24,26,27,28]. Alternatively, four studies also took into account (with different approaches) a time component in the analysis of the correlation [10,16,22,25], while three other studies focused instead on a single region and analyzed the correlation between day-by-day levels of pollutants and correspondent daily COVID-19 data [8,18,19], accounting for a time delay.

However, in all these approaches, there are some critical issues that require specific attention:

(1) Data reliability: It is widely recognized that the official data relevant to COVID-19 were underestimating its actual diffusion [21,29,30,31], and this was particularly true when considering Italy during the first pandemic peak (March–April 2020, ISTAT, https://www.istat.it/it/archivio/245415, last access on 3 November 2021): immediately after the first confirmed case, screening protocols were arranged in an attempt to understand the contagion line, tracing back all contacts of people positive to tests but, after few days, when the recorded cases exceeded tracing possibilities [32], testing started to be performed (according to limited capabilities) only to people showing compatible symptoms with COVID-19. Still, diagnosis capabilities were quickly saturated, and the swab test for COVID-19 was performed for the most severe patients only [33,34].
(2) Confounding factors: As clearly stated in a letter to the editors by Riccò et al. [35], it is possible that, studying the relationship between air pollution and COVID-19 cases, ‘we are observing a correlation rather than a causation’. The use of territorial subdivisions for administrative purposes may force the comparison of areas with significant socio-economic disparities, which is likely to be a much more relevant factor (compared to pollution) when assessing the speed of diffusion of the pandemic. Therefore, Riccò et al. suggested that ‘a more appropriate way in dealing with and understanding the relationship between air pollution and SARS-CoV-2 infection incidence rates may occur comparing geographical areas characterized by similar socio-economic development, but strikingly different environmental status (e.g., highly polluted areas versus those with low pollution levels)’. Some studies [13,14,17,20,21,23,26] did take into account possible confounding factors (as suggested by the literature [29]), such as population density [13,20,26], population age [13,14,20,26], socio-economic status [20,23,26], ethnicity [20], people mobility [17,20], and healthcare resources [20,23].
(3) Lockdown-related biases: As once again stated by Riccò et al. [35], ‘it should be stressed that lockdown measures are significantly affecting air pollutants, reducing their daily concentrations’. The comparison of territories where lockdown measures were established at very different epidemic phases (a widely applied approach, especially in studies relevant to the Italian territory) lacks in meaningfulness, as the impact of complete lockdown measures heavily affects both phenomena under analysis (COVID-19 epidemic and air pollution).

To overcome possible limitations due to these critical issues, we hypothesized the following:

- Diffusion of the pandemic could be inferred by the analysis of georeferenced Emergency Medical Services (EMS) interventions (ambulances dispatches) for respiratory problems, which is an approach we recently proposed and validated [36]. Such indirect data are not biased by testing policies and management strategies, which instead affected consistently the reliability of official data, especially in the very first phases of the pandemic.
- A comparison among areas having very similar territorial and socio-economic characteristics within the Lombardy region could highlight possible differences in relation to pandemic diffusion and air pollutants, thus reducing the effect of confounding factors; in addition, limiting the analysis during the first peak (up to 23 March 2020) could minimize the confounding effects of lockdown measures.

Accordingly, the aim of this study was to explore the possible effects of different indicators of air pollution in facilitating the spread of the COVID-19 epidemic in the Lombardy region (a total area of 23,863.65 km^2^) in Italy during the first period of the pandemic by a novel approach based on the subdivision of the territory in homogeneous areas based on land utilization, and among them, the comparison of the correlations between the mean concentrations of air pollutants computed over different exposure intervals (starting one, six, and twelve months before 31 January 2020), and the speed of the evolution of the pandemic, which was estimated using the daily incidence EMS interventions for respiratory problems over the resident population. To this scope, a Geomatics approach was applied with the use of Geographic Information Systems, which is a set of tools for capturing, storing, checking, manipulating, analyzing, and displaying spatially georeferenced data [37].

## 2. Materials and Methods

### Data Sources and Software

All the information relevant to the administrative limits and to the demographics of the resident population were obtained as open data by the Italian National Institute of Statistics website (ISTAT, Istituto Nazionale di Statistica, https://www.istat.it/it/, accessed on 3 November 2021), which was updated on 1 January 2019. The georeferenced database of EMS ambulances dispatched for respiratory causes on the Lombardy territory within the target time period, from 1 January 2020 to 23 March 2020, was provided after anonymization by AREU (Agenzia Regionale Emergenza/Urgenza), which is the EMS provider in Lombardy. For each record, the following information were available: date and time (of first arrival, of eventual arrival of means for advanced life support, of eventual arrival to hospital), gender and age of the patient, place (category) and coordinates of intervention, intervention outcome (transport to hospital or not), and hospital where the patient was transported.

Data relevant to pollution indicators were obtained as open data by Agenzia Regionale per la Protezione Ambientale–Lombardia (ARPA, Regional Agency for Environmental Protection–Lombardy division, https://www.arpalombardia.it/Pages/Ricerca-Dati-ed-Indicatori.aspx, accessed on 3 November 2021). Pollutant data were collected by 85 stations (not all stations monitored all the pollutants) distributed on the Lombardy territory, monitoring PM_2.5_ and PM _10_, NO and NO_2_, O_3_, CO, ammonia (NH_3_), SO_2_, and benzene (C_6_H_6_), with a time resolution of one hour.

Data about the land use were retrieved from the DUSAF open database, which was managed by the Lombardy region (http://www.geoportale.regione.lombardia.it, accessed on 3 November 2021).

All processing of georeferenced data was performed using QGIS (https://qgis.org, accessed on 3 November 2021), an open-source Geographic Information System released and updated as part of the OsGeo project and community (https://www.osgeo.org/, accessed on 3 November 2021). Database management and statistical analysis were performed using Python libraries (https://www.python.org/, accessed on 3 November 2021).

For all the 1506 municipalities located in the Lombardy region, the percentage of natural, agricultural, industrial, and urban terrain was computed using the DUSAF database; in addition, the mean annual tax contribution of the resident population (as reported by ISTAT) was considered as a socio-economic indicator. Based on these 5 attributes, a K-means clustering algorithm was applied to classify each municipality into 5 territorial classes (from TC0 to TC4, which were manually selected as the most meaningful subdivision after inspecting all the results with varying number of classes from 3 to 7).

To reduce geographical granularity and the related effect of random noise, neighbor municipalities were aggregated to obtain 77 districts of at least 100,000 residents (as described in [36]), resulting in districts with a median (25th–75th) population of 107,724 (100,382–121,096), except for the cities of Milan, Brescia, Monza, and Bergamo (having each a total resident population above 100,000 units) that formed districts themselves. Then, each district was assigned to the TC corresponding to the majority class of at least 75% of its municipalities, where the 75% threshold was empirically considered as meaningful.

For each district *d*, the total number of ambulance dispatches for each day in the period was computed. Starting from these data, to consider the time component (thus indirectly highlighting the speed of the diffusion of the pandemic), the following procedure was:

- A moving average filter with a window of 5 days was applied;
- The exponential data regression in the form $y=e^{\lambda t+q}$ was iteratively computed on a sequence having as its last point the value at 23 March 2020 (last data point available) and as the first point each possible previous day (moving backward up to 1 January 2020); then, the earliest point with a correlation coefficient R^2^ > 0.9 was selected as the starting day (${\bar{t}}_{d}$) of the pandemic diffusion;
- The parameter $\lambda^{d}$ resulting from the exponential regression between (${\bar{t}}_{d}$) and 23 March 2020 was considered as a representative estimate of the speed of the pandemic diffusion for the district *d*.

**Figure 1:**
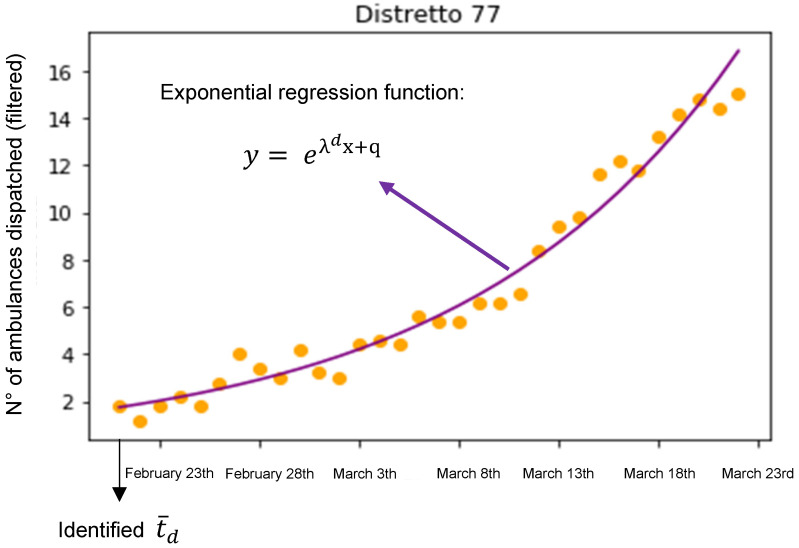
Data representing the daily number of ambulances dispatched in a district, which were filtered with a 5-day moving average and plotted; the day ${\bar{t}}_{d}$ is identified as the earliest day where the exponential regression function ($y=\;e^{\lambda^{d}x+q}$ ) had a correlation coefficient R^2^ > 0.9, and the $\lambda^{d}$ parameter of such a function (computed from ${\bar{t}}_{d}$ ) was considered as the estimator of the speed of the COVID-19 pandemic diffusion.

A graphical representation of the procedure is reported in Figure 1.

**Figure 2:**
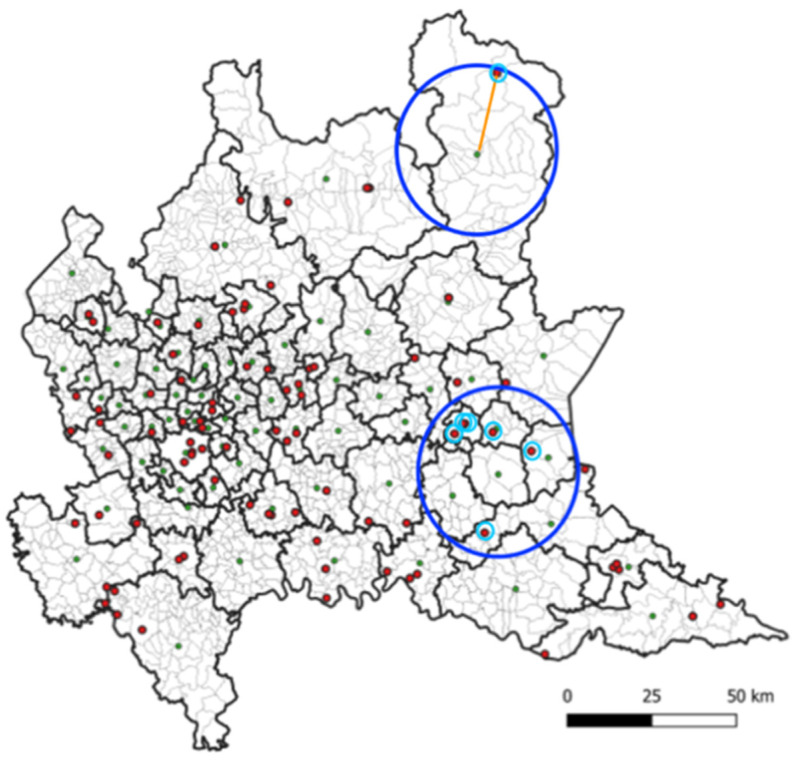
Map of the Lombardy region, indicating the boundaries of the 77 generated districts (see text for details) and their centroid (green dots), together with the position of the air pollutants recording stations (red dots). For each district, the computation of the air pollutants time-series was based on the recorded values of the closest recording stations (maximum 5) in a radius of 25 km around the centroid, with values weighted according to their radial distance to the centroid (see text for details).

As regards air pollution data, for every district d, its centroid was computed, and the five available recording stations (within a 25 km range) closest to the centroid were selected (Figure 2).

For each pollutant, the daily data from these stations were used to compute a spatially weighted average *Pi(t)_d_* based on the Euclidean distance. Finally, for each pollutant *Pi* included in the analysis, from these time series, the mean values over the previous (starting 31 January 2020, before the official COVID-19 first case) 12 months ($\bar{{\bar{P}}_{i}^{d}}12)$, 6 months ($\bar{{\bar{P}}_{i}^{d}}6$), and 1 month ($\bar{{\bar{P}}_{i}^{d}}1)$ were obtained for each district d as reported in the following formula:

(1) $$\bar{P_{i}^{d}}t=\frac{\sum_{m}\frac{\sum_{s}cp_{i,s,m}\;*\;\frac{1}{e_{s}^{d}}}{\sum_{s}\frac{1}{e_{s}^{d}}}}{M}$$

where:

$\bar{P_{i}^{d}}t$ is the average concentration of pollutant *i* in district *d* in the time period *t*, with *i* = 1,…,9 indicating the different considered pollutant (PM_2.5_, PM_10_, NO, NO_2_, O_3_, CO, NH_3_, SO_2_, C_6_H_6_);

*d* = 1,…,77 represents the index of the district;

*t* indicates the considered exposure period (1 month, 6 months, or 12 months starting from 31 January 2020 and going backward in time);

*m* = 1,…,M is the day index in the considered time period (with M = 31 if *t* is 1 month, 183 if *t* is 6 months, or 365 if *t* is 12 months);

*s* = 1,…,S is the index of the considered recording station;

$cp_{i,s,m}$ is the concentration of pollutant *i* measured by the station *s* at day *m*;

$e_{s}^{d}$ is the Euclidian distance between the recording station *s* and the centroid of the district *d*.

**Figure 3:**
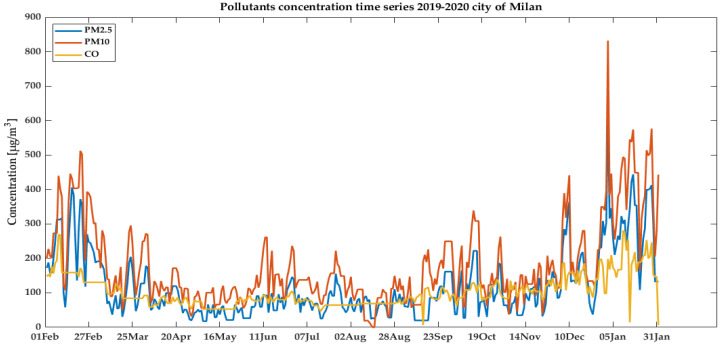
Example of extracted time-series of three pollutants (PM_2.5_, PM_10_, and CO) in the city of Milan for the considered retrospective period of 12 months, from 1 February 2019 to 31 January 2020.

A graphical representation of the time series for PM_2.5_, PM_10_, and CO for the city of Milan in the preceding 12 months is reported in Figure 3.

As a final step, separately for each of the five TC, the values of pandemic diffusion speed $\lambda^{d}$ for each district d∈TC were separately fitted with the exponential regression:

(2) $$\lambda^{d}=e^{\tau *\bar{P_{i}^{d}}t+w}$$

with the corresponding computed values of $\bar{{\bar{P}}_{i}^{d}}12$, $\bar{{\bar{P}}_{i}^{d}}6$, and $\bar{{\bar{P}}_{i}^{d}}1$ (for *i* = 1, …,9) to explore the possible relationship with air pollutants over different retrospective periods of cumulative exposure, thus resulting in 9 × 5 × 3 = 135 different values of R^2^.

## 3. Results

### 3.1. Territorial Subdivision and Clustering

**Figure 4:**
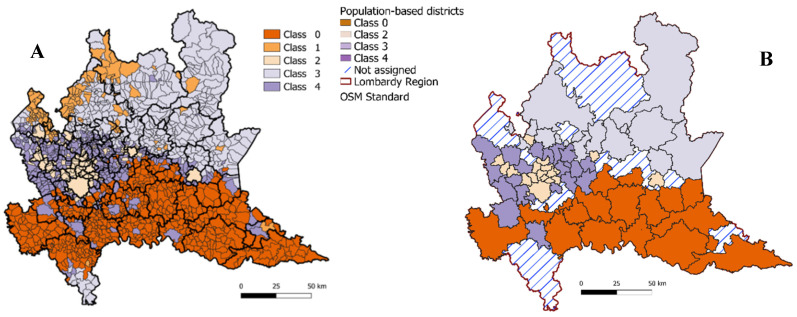
(**A**) Map of Lombardy region with each of the municipality administrative boundaries highlighted (thin black lines), as well as the boundaries of generated 77 districts of approximatively 100,000 residents (bold black lines). The color attributed to each municipality reflects the results of the mapping operation with the corresponding territorial clustering class (see text for details). (**B**) Map of Lombardy region with the boundaries of generated 77 districts superimposed. The color attributed to each district is the result of the mapping operation with the corresponding territorial clustering class (see text for details). Class 0: prevalently agricultural; class 1: mainly natural area with lower income; class 2: mixed land utilization with prevalent urbanized areas; class 3: mainly natural area with higher income; class 4: mixed land utilization with prevalent agriculture/natural areas.

The clustering of municipalities based on the land utilization and mean annual tax contribution of the resident population resulted in a composition of the five TC as described in Table 1, with the associated map of the resulting classification of municipalities over the Lombardy territory reported in Figure 4A: class 0 was prevalently agricultural; classes 1 and 3 reflected mainly natural areas characterized by lower or higher income, respectively; classes 2 and 4 had a mixed land utilization in which urbanized areas or agriculture/natural prevailed, respectively.

Based on the TC of each municipality within each district, out of the 77 districts, for 15, a TC was not assigned (i.e., the threshold of 75% of municipalities belonging to the same TC was not reached, and then, they were discarded from further analysis), 16 were assigned to class 0, 17 were assigned to class 2, 10 were assigned to class 3, and 19 were assigned to class 4, while no district was assigned to class 1. The corresponding map of the districts assigned to the different TC is shown in Figure 4B. These results are coherent with the geography of the territory: class 0 (mainly agricultural) is prevalent in the southern belt corresponding to Pianura Padana (plain and less urbanized); class 2, the most urbanized, is in proximity of the city of Milan, capital of Lombardy, and of the other largest cities; class 3 (mainly not anthropic) is in the northern part of the region, characterized by mountains, while class 4 (most balanced) characterizes the extended hinterland of Milan.

From Table 2, it is possible to evidence that TC0 (mainly agricultural) was characterized by a higher prevalence of ambulance dispatches from 1 January 2020 to 23 March 2020, which was followed closely by TC3 (mainly natural), thus confirming that the epidemic appeared stronger outside the urbanized areas than in more populated clusters (TC2) in proportion to the resident population.

Examining pollutants levels, values relevant to the 1-month observational window appeared higher than those for 6 or 12 months for most pollutants except for O_3_: this can be explained by the origin of the time windows (going backward from 31 January 2020) and the relevant pollution sources in the considered periods.

Considering the different indicators of pollutants concentrations in the different TC, TC0 showed the highest levels of PM_2.5_, PM_10_, NH_3_, SO_2_ in all the observational periods, while TC2 (mainly urbanized) was characterized by the highest concentration of NO, NO_2_, CO, and C_6_H_6_, and TC3 prevailed only for O_3_. These results may reflect the possible main sources of pollution in those classes: in TC0, it was due to agricultural activities, while in TC2, it was due to transportation and heating.

### 3.2. Data Correlations

**Figure 5:**
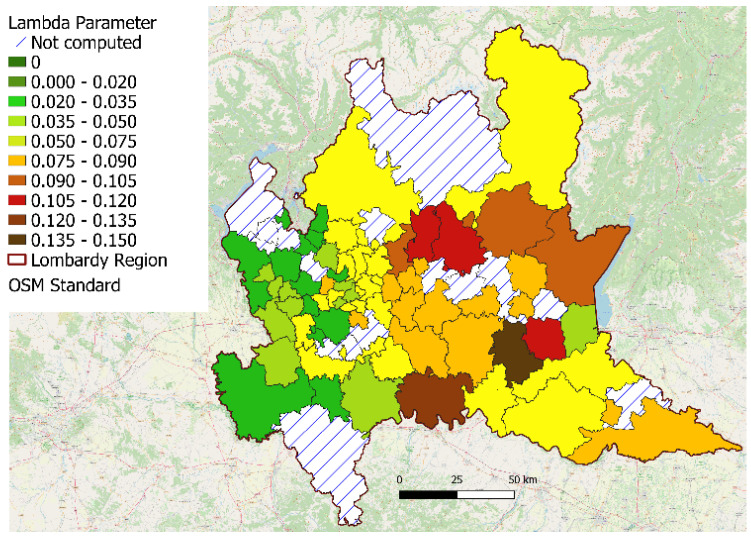
Values of the lambda parameter ($\lambda^{d}$), which was used as an estimator of the speed of COVID-19 diffusion on the basis of ambulances dispatched for respiratory issues (see text for details) for each district of approximately 100,000 residents, mapped on the territory of the Lombardy region (Italy).

In Figure 5, the values of the $\lambda^{d}$ parameter, representing the estimate of the pandemic diffusion speed computed from the ambulance dispatches, are color-coded for each district.

**Figure 6:**
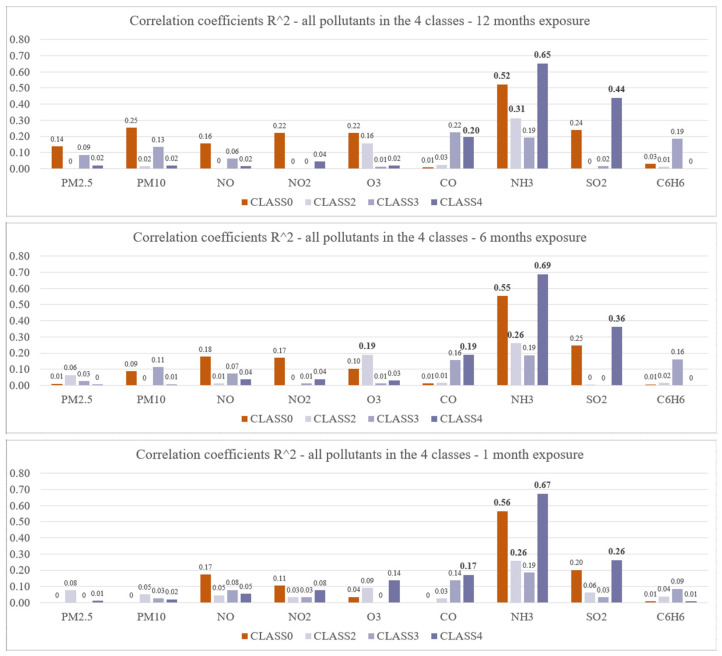
Bar chart representation of all the correlation coefficients between the estimated speed of COVID-19 diffusion and the average concentration of nine different pollutants computed in the previous 12, 6, and 1 months, separately for the four classes representing comparable territorial areas (see text for details); values in bold highlight correlation coefficients with a *p*-value < 0.05. Class 0: prevalently agricultural; Class 2: mixed land utilization with prevalent urbanized areas; Class 3: mainly natural area with higher income; Class 4: mixed land utilization with prevalent agriculture/natural areas.

The class-specific correlation between the speed of diffusion of COVID-19 (estimated from the dispatched ambulances for respiratory issues) and the exposition to pollutants was evaluated on the basis of the correlation coefficient R^2^ for exponential regression ($\lambda^{d}=e^{\tau *\bar{P_{i}^{d}}t+w}$). A complete representation of the results is reported in Figure 6.

The correlation distributions for each TC for each pollutant were examined: Ammonia showed statistically significant values (*p* < 0.05) considering all the exposure time periods (1, 6, and 12 months) in TC0 (mainly agricultural use), TC2 (mainly urbanized), and TC4 (balanced land use). Conversely, CO and SO_2_ showed significant values for all exposure periods but only in TC4, while for O_3_, it was significant for TC2 and 6-months exposure only. Based on these results, as ammonia showed high R^2^ values (the highest value of R^2^ was 0.69 for class 4 at 6 months mean exposure) in different classes, further considerations will be focused on this pollutant only, considering a value of R^2^ > 0.5 as cut-off.

Table 3 summarizes the results of the exponential regression between ammonia concentration indicator and the estimated speed of COVID-19 diffusion, which were measured with the parameter $\lambda^{d}$ computed from the ambulance dispatches in the different TC and exposure time windows.

It is possible to notice how the values of ***τ***, representing the slope of such a correlation, were higher in TC4 compared to TC0.

**Figure 7:**
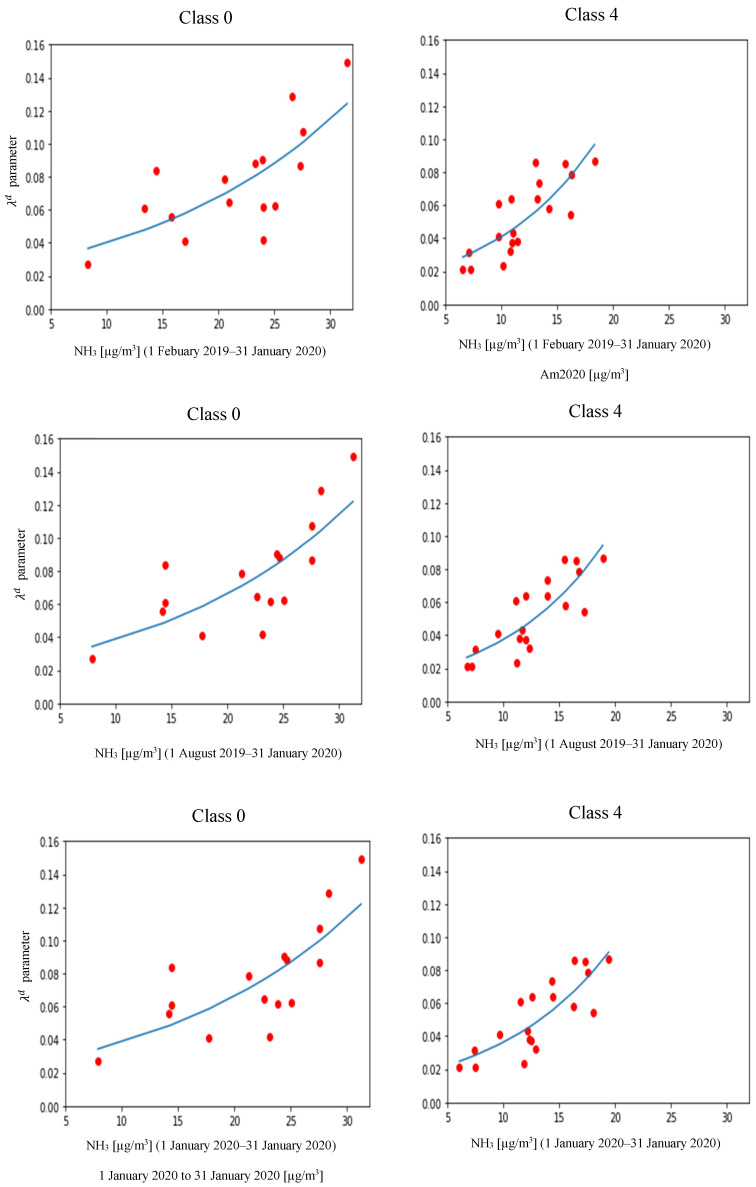
Plots (each red dot refers to one district) of the mean level of ammonia (NH_3_) recorded in a specific time period against the $\lambda^{d}$ parameter (estimation of the speed of diffusion of COVID-19; see text for details) for all districts belonging to the same territorial clustering class (areas comparable from the point of view of territory characteristics, see text for details), and exponential regression of data distribution (blue line). Class 0: prevalently agricultural; Class 4: mixed land utilization with prevalent agriculture/natural areas.

In Figure 7, the different graphs show the computed exponential regressions for ammonia with the speed of COVID-19 diffusion, where each red dot represents the measured combination of pollutant concentration and $\lambda^{d}$ for each district included in the corresponding TC. By comparing TC0 (mainly agricultural) with TC4 (balanced land use), it appears evident how, in the presence of higher values of NH_3_ concentration in TC0, the corresponding values of $\lambda^{d}$ are following the exponential relation, reaching values up to double those observed in TC4.

## 4. Discussion

This is the first study in which the association between the estimated speed of diffusion of COVID-19 and the level of exposure to several pollutant indicators was studied, considering land utilization in the comparison. Interestingly, the strongest association was found for the ammonia indicator.

Specifically, this correlation emerged in TC0, which consisted of prevalently agricultural areas (76.28%,) and TC4, which consisted of a balanced share of different land uses (of which 33.18% was agricultural use). Interestingly, in TC4, the correlation was higher (R^2^ = 0.688) when compared to TC0 (R^2^ = 0.565), and it was associated to values of τ (i.e., describing the slope of the relation between the estimated speed of COVID-19 diffusion and measured level of ammonia indicator) higher by 0.0347, 0.0298, and 0.0279, respectively for 12, 6, and 1 month duration, despite the lower levels of ammonia measured in the same observation periods. This could be due to the higher level of urbanization in TC4, supporting the hypothesis that a higher density of population, in territories where urbanized areas are interleaved with agricultural activities and consequently exposed to NH_3_ pollution, thus creating the conditions of pre-existing fragility, could result in a more evident and faster spreading of COVID-19 due to its high rate of human-to-human transmission. In addition, very similar values were observed comparing the three different observation periods for both TC0 and TC4, thus evidencing how the estimated speed of diffusion was related to the mean level of ammonia indicator recorded in the period, with no difference between the longer (12 months) or shorter (1 month) continuous exposure.

Surprisingly, when considering the prevalence of ambulance dispatches over the considered period, TC0 showed the highest values, followed by TC3 (mainly natural), confirming that the epidemic appeared stronger outside the urbanized areas; hence, in TC3, no specific correlation with pollutant indicators was evidenced, thus suggesting other causes for this phenomenon possibly related to the movement of people (already infected) toward the countryside (i.e., pre-Alps and Alps areas) at the beginning of the pandemic, in addition to the early lockdown established in Lombardy since 9 March 2020.

When comparing the pollution indicators concentrations over the different observation periods, their distribution in the different TC is in line with the expected main sources of pollution. Ammonia concentration was the highest in TC0, with lower values measured in TC4; high values were recorded also in TC2 (strongly urbanized), hence with weaker correlation with the speed of diffusion estimated by ambulance dispatches. A possible explanation for this observation is that strongly urbanized areas are characterized by other unidentified drivers and pollution sources with a different impact on our indicator, generating different mechanisms that hid the trends observed in TC0 and TC4.

In addition to these considerations, although it is well known that NH_3_ has a strong impact on the generation of other pollutants, such as SO_2_, NO, NO_2_, and, especially, secondary particulate matter [38,39], in the proposed analysis weaker correlations were found with these derived pollutant indicators.

Our results are coherent with the analysis conducted by ARPA, which identified in agricultural activity the main source (85%) of ammonia production (https://www.arpalombardia.it/Pages/Aria/Aria-Progetti/Progetto-Ammoniaca.aspx, accessed on 3 November 2021), and with scientific literature on the topic [40,41,42,43]. Indeed, the emission of NH_3_ from animal husbandry is recognized as the main source of NH_3_ in the atmosphere [44], mainly originating from the microbial decomposition of nitrogen-containing organic matter in manure. In both Europe and the United States, about 80% of total NH_3_ emissions comes from animal waste [45].

The effects of NH_3_ on the organisms depend on the concentration and duration of the stimulation. Remodeling of the tracheal epithelium, the barrier between the body and the environment, can originate from the stimulation of external gases [46] and result in smooth muscle hyperplasia, pulmonary fibrosis, basal layer thickening, cell composition changes, and inflammatory cell infiltration. NH_3_ exposure leads to a loss of cilia or to the production of more mucus covered on the basal layer of tracheal cilia, and it also could cause chronic respiratory diseases and significantly aggravate the symptoms of emphysema and asthma [47].

A recent study conducted in pigs, an animal model closely related to humans in terms of metabolism, anatomy, and physiology [48], identified that NH_3_ could break down the mucosal barrier of the respiratory tract, induce oxidative stress and inflammation in pig trachea, reduce the activity of microtubules, and disrupt the balance of solute carrier (SLC) transporters [49]. Oxidative stress represents an imbalance between the production of reactive oxygen species (ROS) and antioxidant defenses, causing damage to crucial biomolecules and cells, and affecting the entire organisms [50], leading to cell death by the destruction of cellular components [51]. Indeed, the mechanisms of air pollution-induced health effects involve oxidative stress and inflammation: particulate matter (PM), ozone, nitrogen oxides, and transition metals are potent oxidants or able to generate ROS. Oxidative stress is one of the toxicity mechanisms also associated to NH_3_ [52,53].

Interestingly, SARS-CoV-2 infection pathogenesis has been related to oxidative stress by ROS as a response to aggression, with a perpetuation of the cytokine storm cycle, blood-clotting mechanism, and exacerbating hypoxia in those patients that require hospitalization [54,55].

As several studies highlighted the strategy of some viruses to alter the redox balance of a cell in order to survive, inducing oxidative stress in order to facilitate their replication inside the cell [56], on the basis of our results, we could speculate that continuous exposure to NH_3_ pollutant could have generated a pathophysiological substrate of inflammatory status together with oxidative stress in favor of virus replication and thus facilitated the further manifestation of clinical conditions that required the intervention of EMS with higher levels associated to faster spreading of the virus. In urbanized areas where agricultural activity was also present, this potential inflammatory condition, together with the greater residential density, facilitated the virus transmission.

While results are various across recently published studies, most of them agree on the identification of a correlation between COVID-19 data and PM_2.5_ (i.e., infections [8,13,14,15,18,22,24,26,28] and casualties [8,12,14,15,18,21,23,25,26,28]), PM_10_ (i.e., infections [8,11,15,17,18,22,24,26] and casualties [8,12,15,18,23,25,26]) and NO_2_ (i.e., infections [8,13,14,16,19,22,24,26,28] and casualties [8,12,14,19,20,26,27,28]). However, these correlations were not evident in our analyses.

Since the key point of our novel approach was to make a comparison of results among areas with similar characteristics in terms of land use, it is realistic to hypothesize that the previously identified correlation between PM and COVID-19 diffusion could have an indirect origin: areas that were more densely inhabited were struck harder by the COVID-19 epidemic in terms of diffusion because of the anthropization of the territory and, as these areas are characterized by a much higher concentration of PM, the two phenomena appeared correlated but without a direct risk association. This hypothesis is further corroborated by the use, in literature, of only official diagnostic data, considering that in metropolitan areas, compared to rural ones, all the infrastructures and protocols for testing could be organized faster and with higher efficiency.

Moreover, the study of the impact of air pollution on COVID-19 diffusion and its lethality is strictly related with the investigation on the mechanisms of airborne transmission of the SARS-CoV-2 virus, which is consistently debated in current research [12,57,58,59,60,61,62,63] in light of its importance in the identification of strategies for limiting the spreading of the pandemic. It is known that virus-laden aerosol could interact with atmospheric particles, creating clusters acting as carriers, thus enhancing the persistence of viruses in the atmosphere [64,65], and therefore, it was hypothesized that the same mechanism could apply to SARS-CoV-2 virus [6,61], which is a hypothesis that some research groups consider enforced by the resulting correlation between PM concentrations and COVID-19 data [11,17,18,22] and the identification of SARS-CoV-2 RNA on particulate matter [66,67]. However, more recent studies stated that there is currently not enough evidence to prove this mechanism to be a factor in the pandemic diffusion [10,29,63].

Based on our exploratory research, we agree with this more recent interpretation and suggest the possible role of intensive farming (and relevant NH_3_ pollution), especially in proximity of urbanized areas, in creating a pre-existing fragility that could result in a facilitating factor for the spreading of viruses such as SARS-CoV-2 and generating worse clinical outcomes in COVID-19 patients [6,29,68]. However, the evaluation of the possible pathophysiological mechanisms behind ammonia exposure and COVID-19 was beyond the aim of our study, and specific research will be needed to further corroborate our hypothesis.

The lack of reliable and timely official data related to the COVID-19 epidemic led to the use of the ambulance dispatches as an unbiased estimator of the virus spreading, although it was not possible to distinguish between interventions actually related to COVID-19 diagnosis and other respiratory issues. However, we recently demonstrated the effective relation between this indicator and mortality in Lombardy [36]. 

The adopted ecological design, based on grouping adjacent municipalities into districts of approximately 100,000 residents from which to measure the number of ambulance dispatches, represented a compromise between spatial resolution and the need of having enough events to trigger a visible increase of cases in a specific cluster. As such, a different territorial subdivision (using different number of residents, or applying different criteria), could result in different municipalities composing a district, and as such potentially impacting on the territorial clustering of the district, oand on the final results. However, as shown in [36], the applied methodology could be considered sufficiently robust to spatial variations in the subdivision of the territory, thus providing curves representing the daily number of ambulances dispatched in a district with an exponential trend morphology. 

The use of ground stations, not uniformly distributed across the territory, to measure air pollutants in each district implies a strong approximation, as local phenomena nearby the stations as well as geographical factors could impact on the measured values. The use of satellite imagery, such as data from the Copernicus project, could provide more accurate measurements. In addition, in the computation of the mean concentration of the pollutant indicator over temporal periods, the underlying assumption was that residential population was always exposed to these levels, which might not always correspond to reality due to possible mobility across districts.

Despite the apparent coherence in the obtained results, we cannot exclude the presence of several confounding factors in the observed phenomena, which could lead to alternative explanations. As already mentioned, we did not consider the possible movement flows of people from the urbanized areas toward natural areas before and during the lockdown, possibly caused by the perception of risk at the arrival of the epidemic and its modifications during the epidemic curve, also potentially in differentiated phases of the disease (asymptomatic or symptomatic period). This limitation, thus resulting in people being exposed to pollutant levels different over time to what was hypothesized in our approach, should be tackled in further studies where mobility data could be available. Similarly, the possible role of animals as carriers to facilitate the spreading in rural and farming areas cannot be a priori excluded.

## 5. Conclusions

In this study a novel method was proposed and applied to explore the role of air pollution indicators on the estimated speed of diffusion of the COVID-19 pandemic during the first outbreak (1 January 2020 to 23 March 2020), with specific reference to the territory of Lombardy region, Italy. The main novelties were related to the following:

- The reliability of analyzed data, by considering the number of ambulances dispatches for respiratory issues;
- The management of confounding factors, by comparing only territories with similar land-use characteristics;
- Considering the time component, by focusing on the initial spread of the disease, to avoid biases introduced by effect of lockdown measures;
- The method to estimate the speed of diffusion, by computing the slope of the exponential interpolation of daily data rather than using cumulated data.

For the first time, a risk association between ammonia indicator levels and estimated speed of SARS-CoV-2 virus diffusion was found in conjunction with mainly agricultural or mixed (urbanized and agricultural) land utilization. From these results, we could speculate that oxidative stress, as one of the toxicity mechanisms associated to NH_3_, could have contributed to a pre-existing fragility due to inflammatory status in residents of those areas, thus facilitating virus replication and further worsening of clinical conditions that required the intervention of EMS.

Our study underlines the importance of tracking ammonia concentration indicators together with other pollutants as well as the need to thoroughly investigate the possible pathophysiological mechanisms behind ammonia exposure and COVID-19.

The possible role of intensive farming (and relevant NH_3_ pollution exposure), especially in proximity of urbanized areas, in creating a pre-existing fragility able to facilitate the spread of viruses such as SARS-CoV-2, should be carefully investigated in the future. Further analyses of the data relevant to the following waves of contagion could contribute to a better understanding of the robustness of the observed risk association.

## Figures and Tables

**Table 1:** Composition of the different territorial clustering classes (TC) with relation to the five considered attributes.

TC Number	% Urbanized Area	% Industrial Area	% Agricultural Area	% Natural Area	Mean Tax Contribution (€)
0	7.97	4.6	76.28	11.21	15,332.84
1	8.47	1.23	3.51	86.76	10,589.85
2	45.05	16.9	17.02	20.99	18,124.67
3	6.46	1.52	2.84	89.12	15,513.17
4	23.21	10.53	33.18	33.13	17,714.22

**Table 2:** Aggregated data distribution, expressed as median (25th–75th percentiles), for the four clustering classes TC (see text for details), reporting the cumulated prevalence of ambulances dispatched (normalized by 100,000 residents) in the considered time period (1 January 2020–23 March 2020), and the values distribution generated by the different districts composing each clustering class, together with the values distribution of all the measurements for each pollutant in the three considered time windows (12 months, 6 months, 1 month).

		Class 0	Class 2	Class 3	Class 4
Ambulances dispatched/100k residents (1 January 2020–23 March 2020)	Total distribution in districts	699.6 664.8 (561.1–778.1)	437.5 394.7 (340.9–473.4)	650.1 587.7 (469.9–737)	430.7 393 (341.1–503.3)
PM_2.5_ [µg/m^3^]	12 months 6 months 1 month	18 (11–31) 21 (13–35) 51 (36–64)	17 (11–28) 18 (12–32) 48 (35–62)	14 (7–24) 12 (5–24) 22 (4–39.25)	16 (11–27) 17 (11–30) 44 (33–57)
PM_10_ [µg/m^3^]	12 months 6 months 1 month	27 (18–42) 29 (20–46) 62 (47–77.5)	24 (17–35) 26 (17–41) 59 (44–75)	19 (11–30) 20 (10–30) 34 (18.5–52)	24 (17–37) 25 (17–40) 57 (42–72)
NO [µg/m^3^]	12 months 6 months 1 month	26 (14–52) 32 (16–65) 80 (53–117)	53 (29–104) 68 (34–131) 164 (101–256)	20 (11–41) 25 (13–53) 59 (29–98)	31 (17–64) 39 (20–84) 114 (66–185)
NO_2_ [µg/m^3^]	12 months 6 months 1 month	20 (11–33) 23 (13–35) 39 (31–48)	37 (22–56) 40 (24–58) 64 (49–80)	16 (9–29) 19 (10–32) 36 (22–50)	23 (13–39) 27 (15–41) 48 (36–64)
O_3_ [µg/m^3^]	12 months 6 months 1 month	39 (10–74) 20 (4–50) 4 (2–10)	40 (8–75) 18 (6–53) 6 (5–10)	58 (26–87) 38 (13–69) 21 (7–54)	40 (10–74) 19 (6–51) 6 (3–11)
CO [mg/m^3^]	12 months 6 months 1 month	0.3 (0.2–0.6) 0.5 (0.3–0.7) 0.9 (0.7–1.1)	0.6 (0.3–0.9) 0.7 (0.4–1) 1.2 (0.8–1.6)	0.3 (0.2–0.4) 0.3 (0.2–0.4) 0.4 (0.2–0.6)	0.4 (0.3–0.7) 0.5 (0.3–0.8) 1 (0.7–1.3)
NH_3_ [µg/m^3^]	12 months 6 months 1 month	17 (7–40) 17 (6–37) 22 (6–43)	13 (6–18) 14 (12–16) 15 (13–16)	3 (1–6) 3 (0–5) 2 (0–7)	4 (2–8) 3 (2–7) 3 (2–5)
SO_2_ [µg/m^3^]	12 months 6 months 1 month	2.9 (1.7–4.1) 2.8 (1.7–4) 2.9 (1.9–3.8)	2.2 (1.3–3.5) 2.5 (1.5–3.8) 3.6 (1.9–6)	1.1 (0.7–1.9) 1.5 (0.9–2.4) 2.5 (1.6–3.6)	2 (1.2–3) 2.3 (1.5–3.2) 3 (2.3–4)
C_6_H_6_ [µg/m^3^]	12 months 6 months 1 month	0.4 (0.2–0.8) 0.5 (0.3–1.3) 2 (1.6–2.5)	0.9 (0.5–1.5) 1.1 (0.6–1.9) 2.4 (1.5–3.7)	0.5 (0.3–1.5) 0.9 (0.3–2.1) 1.9 (1.4–2.7)	0.4 (0.2–0.9) 0.5 (0.3–1) 1.6 (1.2–2.2)

**Table 3:** Results of exponential regression between ammonia concentration indicators and the estimated speed of COVID-19 diffusion, measured with the parameter *λ^d^* computed from the ambulance dispatches, in the different TCs and exposure time windows where R^2^ was higher than 0.5; the τ parameter represents the slope of this exponential regression.

Territorial Class	Exposure Time Period [Months]	τ	R^2^ Exp Regression
Class 4	12	0.0943	0.652
Class 4	6	0.0968	0.688
Class 4	1	0.0922	0.674
Class 0	12	0.0596	0.523
Class 0	6	0.067	0.553
Class 0	1	0.0643	0.565

## Data Availability

All data available on request.
